# Quantitative EEG features during the first day correlate to clinical outcome in perinatal asphyxia

**DOI:** 10.1038/s41390-024-03235-y

**Published:** 2024-05-14

**Authors:** Anna Tuiskula, Alexey S. Pospelov, Päivi Nevalainen, Saeed Montazeri, Marjo Metsäranta, Leena Haataja, Nathan Stevenson, Anton Tokariev, Sampsa Vanhatalo

**Affiliations:** 1 Department of Pediatrics, Children’s Hospital, University of Helsinki and Helsinki University Hospital, Helsinki, Finland; 2 BABA Center, Pediatric Research Center, University of Helsinki and Helsinki University Hospital, Helsinki, Finland; 3 Department of Physiology, University of Helsinki, Helsinki, Finland; 4 Department of Clinical Neurophysiology, Children’s Hospital, HUS Diagnostic Center, and Epilepsia Helsinki, full member of ERN EpiCare University of Helsinki and Helsinki University Hospital, Helsinki, Finland; 5 Department of Pediatric Neurology, Children’s Hospital, University of Helsinki and Helsinki University Hospital, Helsinki, Finland; 6 Brain Modelling Group, QIMR Berghofer Medical Research Institute, Brisbane, QLD Australia

## Abstract

**Objective:**

To assess whether computational electroencephalogram (EEG) measures during the first day of life correlate to clinical outcomes in infants with perinatal asphyxia with or without hypoxic-ischemic encephalopathy (HIE).

**Methods:**

We analyzed four-channel EEG monitoring data from 91 newborn infants after perinatal asphyxia. Altogether 42 automatically computed amplitude- and synchrony-related EEG features were extracted as 2-hourly average at very early (6 h) and early (24 h) postnatal age; they were correlated to the severity of HIE in all infants, and to four clinical outcomes available in a subcohort of 40 newborns: time to full oral feeding (nasogastric tube NGT), neonatal brain MRI, Hammersmith Infant Neurological Examination (HINE) at three months, and Griffiths Scales at two years.

**Results:**

At 6 h, altogether 14 (33%) EEG features correlated significantly to the HIE grade ([r]= 0.39−0.61, *p* < 0.05), and one feature correlated to NGT ([r]= 0.50). At 24 h, altogether 13 (31%) EEG features correlated significantly to the HIE grade ([r]= 0.39−0.56), six features correlated to NGT ([r]= 0.36−0.49) and HINE ([r]= 0.39−0.61), while no features correlated to MRI or Griffiths Scales.

**Conclusions:**

Our results show that the automatically computed measures of early cortical activity may provide outcome biomarkers for clinical and research purposes.

**Impact:**

- The early EEG background and its recovery after perinatal asphyxia reflect initial severity of encephalopathy and its clinical recovery, respectively.
- Computational EEG features from the early hours of life show robust correlations to HIE grades and to early clinical outcomes.
- Computational EEG features may have potential to be used as cortical activity biomarkers in early hours after perinatal asphyxia.

## Introduction

Perinatal asphyxia remains a significant neonatal neurological adversity that requires assessment and intervention during neonatal care, and results in an increased risk of lifelong neurodevelopmental problems.^1–6^ Clinical symptoms define the diagnostic grades of hypoxic-ischemic encephalopathy (HIE), however continuous brain monitoring is recommended because it provides a fair prediction of neurodevelopmental outcome^7–9^ especially in combination with brain magnetic resonance imaging (MRI).^10,11^ The bedside assessment of cortical functional recovery is currently based on visual review of the electroencephalography (EEG) signals,^12^ which is most often done using a time and amplitude-compressed trend, amplitude integrated EEG (aEEG).^13^

Visual review of either raw EEG signals or aEEG trend (hereafter jointly called (a)EEG) is qualitative and inherently subjective.^14–18^ The visual review also needs efforts put into expert training that are not available in most neonatal intensive care units (NICU) worldwide.^19^ Therefore, various computational means have been developed, with a collective aim to support early neonatal (a)EEG monitoring by facilitating routine bedside review, or to benchmark clinical trials. It is a common experience that the worst situations are relatively easy to identify, but there is a notable challenge in assessing majority of infants with milder clinical presentation. In particular, infants presenting with clinically categorized HIE as mild to moderate (grade 1 or 2, HIE1 or HIE2, respectively) may show widely ranging neurological outcomes, from typical to severe impairments.^1,2,4,5,20^

It is reasonable to assume that the widely differing outcomes within and between HIE grades are represented in the latent characteristics of the cortical recovery measured with (a)EEG monitoring in ways that could escape visual recognition of the raw EEG signals or aEEG trends. Indeed, several recent studies have shown that computational EEG measures may show feature-related differences between HIE categories^21–23^ or correlate with clinical outcomes.^24–26^ Those features are typically chosen heuristically, measuring EEG’s spectral^21^ or amplitude content,^22^ cross-frequency interactions,^27^ non-linear characteristics,^28^ or scaling properties in the bursting behavior.^29^

Here, we aimed to identify objective, quantitative features of the EEG signal, i.e., potential functional biomarkers of asphyxia severity, that could complement early clinical evaluation of the infant with perinatal asphyxia. We extracted computational EEG features from two time points during the first day of life and validated these EEG features by correlating them to clinical HIE classification and early neurological outcome.

## Methods

**Fig. 1:**
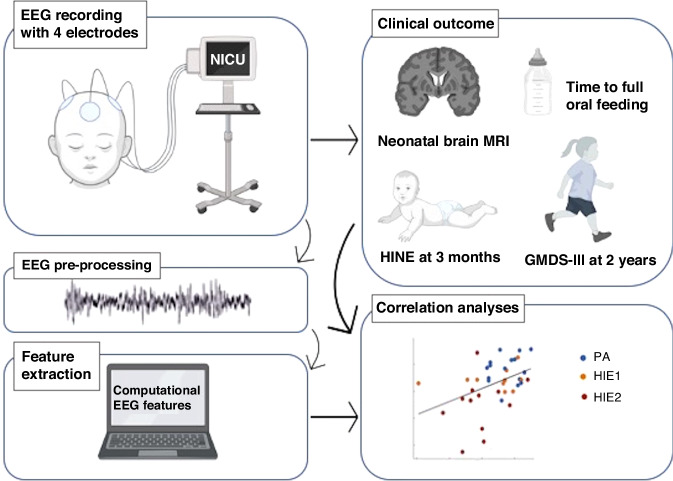
The study overview. Infants with perinatal asphyxia were monitored with a 4-channel (a)EEG in the NICU. After pre-processing, a set of computational EEG features were extracted, to be correlated to the clinical outcomes.

An overview of the present study is shown in the Fig. 1. Long-term (a)EEG monitoring with four scalp electrodes was performed in NICU for term infants with perinatal asphyxia. The computational EEG features were estimated and correlated to several clinical outcomes. This study was approved by the hospital district of Helsinki and Uusimaa (HUS/1331/2016). Parents of the participants gave their informed consent for the research and publication of the results.

### Study subjects and clinical grouping

The present study included 91 infants with perinatal asphyxia, out of which 40 infants had outcome (cohort 1) assessments, and the rest were included to expand the range of HIE severity (cohort 2). Both cohorts recruited full-term infants with clinical signs of perinatal asphyxia, no other reason for distress at birth and at least one of the following: umbilical arterial cord pH below 7.10, a 1-min Apgar score not exceeding 6, need for assisted ventilation or cardiopulmonary resuscitation at birth. Based on severity of clinical symptoms,^30^ infants were subcategorized in to four groups: perinatal asphyxia without HIE (later referred as PA), mild HIE (HIE1), moderate HIE (HIE2) and severe HIE (HIE3), later referred to as HIE groups. Infants were collated from two previously published cohorts: Cohort 1^31^ contributed 40 infants (PA *n* = 18, HIE1 n = 10, HIE2 *n* = 12) and a more detailed clinical characterization, including clinical outcome measures (see below). Cohort 2^32,33^ complemented the dataset with more severe cases (HIE2 *n* = 40, HIE3 *n* = 11) to allow a more balanced correlation between HIE grades and EEG features. An experienced neonatologist (MM) uniformly assessed the clinical HIE categorization for both cohorts by reviewing the medical records and according to the worst assessment (the first day for cohort 1, and days 1−4 for cohort 2). Decision of therapeutic hypothermia was made according to clinical guidelines.^34^ Patients were treated with anticonvulsants if clinically indicated (Table S3).

### Clinical outcome measures

Prospectively collected clinical outcome data was available for 40 infants (18/PA, 10/HIE1, 12/HIE2; see also Fig. S1 for details). To depict early clinical outcome we used time to full oral feeding (number of days, continuous variable) as a neonatal clinical marker of recovery,^35^ visually determined neonatal brain MRI score^36^ (continuous variable ranging 0−57) and Hammersmith Infant Neurological Examination at three months of age (HINE)^37^ (continuous variable ranging 0−78). At two years of age outcome was assessed using standardized developmental quotient (DQ) score of Griffiths Scales of Child Development, 3rd Edition (GMDS-III)^38^ (continuous variable ranging 50-150).

### EEG recordings

**Fig. 2:**
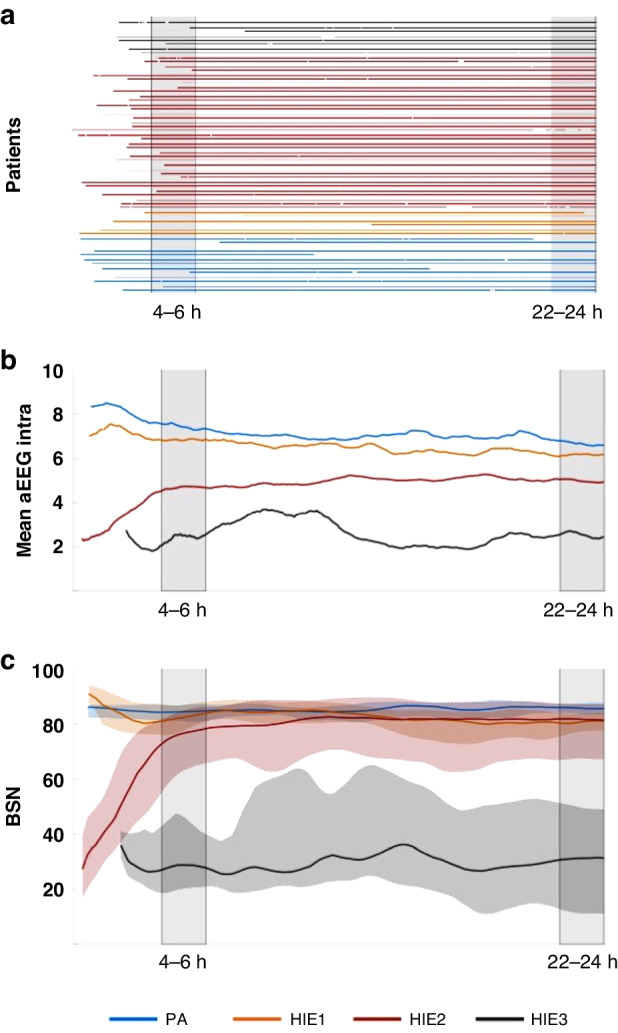
Summary of the (a)EEG-dataset and an example of a temporal dynamics of two individual EEG features. **a** A temporal summary of the EEG data available during the first 24 h of life shown for each patient, as colored by the HIE group. The vertical gray shades indicate time periods 4−6 h and 22−24 h, respectively, that were used in further analyses. **b** Temporal dynamics of intrahemispheric mean aEEG over the first 24 h, shown as the median value in each HIE grade (average smoothing over 2 h). At the group level, the extreme groups are clearly separable throughout the recording, while the other groups are less distinctive, especially when considering the intra-group variability. **c** The temporal dynamics of the median value of BSN in different HIE grades. Shaded areas represent interquartile intervals. Infants with PA and HIE1 generally have higher BSN values very early on, while infants with HIE2 start with a low BSN that recovers towards more normal levels during the first day of life. In contrast, the HIE3 is associated with low values of BSN without clear recovery.

The long-term EEG recordings were performed with a NicOne EEG system (Carefusion/Natus, Madison, WI) using four (F3, F4, P3, and P4) recording electrodes and a recording reference electrode near frontal midline. Only the standard bipolar derivations (F3-P3, F4-P4, F3-F4, and P3-P4) were used in the present study. The recordings were started during the first hours of life (see Fig. 2a), and the timing of (a)EEG recording was comparable between HIE groups. The (a)EEG monitoring data from the first 24 h of life was imported into Matlab (Mathworks) using EDF format as needed, and further processing was done using custom-built algorithms.

### EEG preprocessing

The raw EEG signals were first bandpass filtered within 0.4−30 Hz using a pair of high-pass and low pass Butterworth filters with the corresponding cut-offs, then re-sampled to 250 Hz from the original recordings with variable sampling frequencies (Fs = 250−2 kHz). The artefacts were identified in four-seconds-long epochs with two-seconds-long overlap using a previously published machine learning-based classifier.^39^ The corresponding segment of EEG was included if probability >0.75 for being classified as clean EEG (i.e., non-artefact). There was an overall impression that the worse HIE grades had somewhat higher incidence of artefacts (see Fig. S1C), however visual assessment of artefact rejections suggested that rejections were not a cause of meaningful bias. In addition, we used median values over relatively long (2 h) EEG segments that render the results more robust to brief artefact rejections.

### Computation of EEG features

We computed 42 features from the EEG derivations as mean values over each two minutes-long epochs with 1 min overlap. This yielded us original 1-min resolution which was later compressed in time to median values over 2-h segments. The feature set was chosen to represent a wide selection of EEG characteristics that have been shown earlier to correlate with EEG background or clinical course. The features can be generally categorized spatially intrahemispheric, interhemispheric, local, or global^16,40^ or functionally (measures of amplitude, spectra, cross-frequency interaction, or connectivity); for more details, see Supplementary material, Table S1. For the intrahemispheric measures, we computed average of the feature from both hemispheres (i.e., F3-P3 and F4-P4 derivations). In addition to the *N* = 41 individual features, we also computed a more interpretative feature, Brain State of the Newborn (BSN)^41,42^ which is a deep learning -based continuous index for the EEG background activity ranging from 0 to 100. It was added to the feature set to allow systematic assessment of relations between BSN, individual features, and clinical outcomes.

### Analysis strategy for correlating EEG features to clinical outcomes

**Fig. 3:**
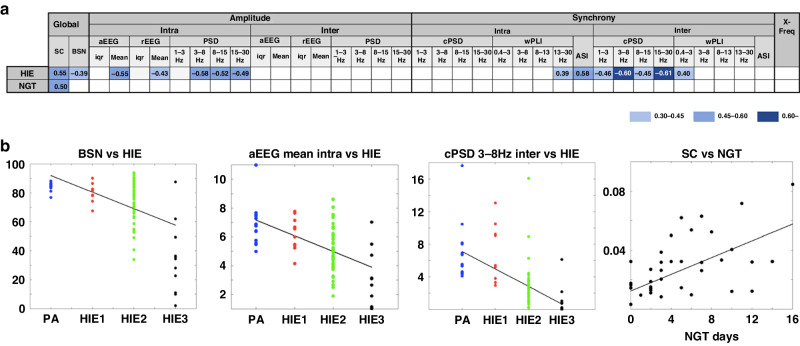
Correlations of EEG features to clinical outcomes at 6 h. **a** The matrix presents significant correlations between EEG features at 6 h and clinical outcomes. The EEG features are grouped according to their underlying neurophysiological mechanisms (global, amplitude, synchrony, and cross-frequency, respectively). The matrix shows only findings that remain significant after correction for multiple comparisons (Benjamini-Hochberg) in each group. Bold fonts show significant correlations after correction for multiple comparisons (Benjamini-Hochberg) over all *N* = 42 EEG features. Color indicates strength of correlation coefficient. The results after spatial EEG features grouping are shown in Fig. S3. For explanations of EEG features, see Table S1. **b** The scatter plots show comparisons between selected EEG features and clinical characteristics at 6 h. BSN = brain state of the newborn, cPSD = cross power spectral density, SC = suppression curve, NGT days = days with nasogastric tube ( = time to full oral feeding).

**Fig. 4:**
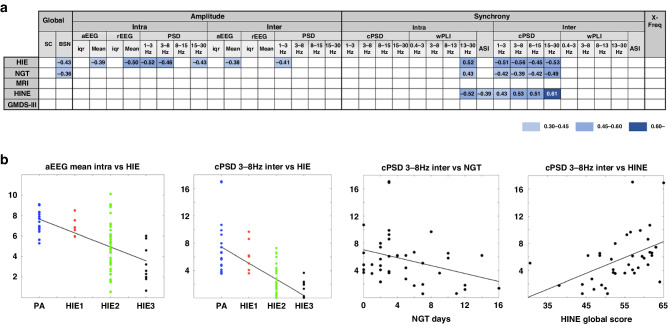
Correlations of EEG features to clinical outcomes at 24 h. **a** The matrix presents significant correlations between EEG features at 24 h and clinical outcomes. The EEG features are grouped according to their underlying neurophysiological mechanisms (global, amplitude, synchrony, and cross-frequency, respectively). The matrix shows only findings that remain significant after correction for multiple comparisons (Benjamini-Hochberg) in each group. Bold fonts show significant correlations after correction for multiple comparisons (Benjamini-Hochberg) over all *N* = 42 EEG features. Color indicates strength of correlation coefficient. The results after spatial EEG features grouping are shown in Fig. S3. For explanations of EEG features, see Table S1. **b** The scatter plots show comparisons between selected EEG features and clinical characteristics at 24 h. BSN = brain state of the newborn, cPSD = cross power spectral density, SC = suppression curve, NGT days = days with nasogastric tube ( = time to full oral feeding), HINE = Hammersmith Infant Neurological Examination.

After the initial inspection of the full time courses and featureXfeature correlations, we continued with clinically motivated analysis paths using EEG features from two discrete time points (postnatal ages). For the very early time point (6 h), we reasoned that the EEG is primarily needed for an immediate assessment of the infants encephalopathy grade (HIE) and the very early outcome measure (NGT). For the little later, early time point (24 h), we reasoned that the EEG is potentially needed for correlating to all of the five outcome measures available in our dataset. Both time points included the same set of *N* = 42 EEG features, calculated from the respective time intervals (average of 4−6 h and 22−24 h of age). As graphically depicted in Fig. S4, each outcome was assessed as an independent question, and corrections for multiple comparisons by Benjamin-Hochberg were done according to three different analytic approaches. First, all the EEG features were correlated to the given clinical outcome (*N* = 42 comparisons). Second, the EEG features were ordered spatially into four groups (*N* = 2−17 comparisons per group). Third, the EEG features were ordered by neuronal mechanisms into four groups (*N* = 2−18 comparisons per group). Results of these analyses are shown in Figs. 3, 4 and Fig. S5. For a full transparency, all (uncorrected) correlation results are shown in Table S2 and visually summarized in Fig. S3.

### Statistical analyses

Correlations between the EEG features and clinical data were computed using the default Matlab function to calculate Spearman correlation coefficients. Statistical significance for the continuous nonparametric outcome variables was calculated with Kruskal–Wallis. Multiple comparisons were corrected by using Benjamin-Hochberg method according to the analysis strategy described above and graphically depicted in Fig. S4. Significance level was set at *p* < 0.05.

## Results

### Clinical outcome

Regarding cohort 1, clinical characteristics such as neonatal brain MRI scoring and HINE total score at three months have been previously reported.^31^ The majority of infants 35/40 (88%) needed feeding with nasogastric tube (NGT). Median (IQR) time to full oral feeding was 2 (0−3) days for PA, 4 (3−7) for HIE1 and 8 (5−12) for HIE2 (*p* < 0.001). At two years GMDS-III developmental quotient (DQ) of all the infants in cohort 1 was within normal range. There were no significant differences between HIE groups: median (IQR) DQ was 107 (104−113) for PA, 107 (98−116) for HIE1 and 104 (96−115) for HIE2 (*p* = 0.833).

### EEG features

Figure 2a summarizes the hours of EEG available in each infant, and examples of the temporally changing trends of EEG features (intrahemispheric mean aEEG and BSN) are shown in Fig. 2b, c. These group average trends show clearly that the mean values in patient groups exhibit distinct trajectories throughout the first day of life. However, there is a significant overlap between patient groups when considering the intragroup variability (Fig. 2b). The HIE2 group may also show a clear rapid recovery from an initial overlap with the HIE3 group and later overlap with the groups of milder grades (HIE1, PA; Fig. 2c). These observations suggest directly that computational features need to be considered dynamically, and at least they cannot be collapsed over longer than maximum of few hours epochs. As a baseline, we also computed mutual correlations in the full feature set. It shows expectedly, that several computational features exhibit substantial mutual correlations, in particular when they measure the same overall neuronal mechanisms (e.g., amplitude- or synchrony -based features; See the heatmaps in Fig. S2). Based on these observations and clinical reasoning (see above), our correlation analyses of EEG vs clinical outcomes used 2-hourly averages from two postnatal time points: at 6 h (very early) and at 24 h (early).

### Correlation of EEG features to clinical outcome

A summary of all correlations after correction for multiple comparisons at 6 h of age is presented in Fig. 3 and at 24 h of age in Fig. 4. The majority of features that correlated to clinical HIE grade were related to signal amplitude or power, spatial correlations (cPSD, wPLI; see Table S1), or the BSN.

#### Correlations at 6 h of age

Altogether 14 (33%) EEG features correlated significantly to the HIE grade ([r]= 0.39−0.61, *p* < 0.05). The most prominent findings included global assessment of continuity (SC; *r* = 0.55), background level (BSN, *r* = -0.39), intrahemispheric measures of amplitude (aEEG, rEEG, PSD) as well as several synchrony measures from the intrahemispheric comparisons (ASI, wPLI) as well as interhemispheric comparisons (cPSD, wPLI). Only the global assessment of continuity correlated to NGT (SC, *r* = 0.50). Notably, measure of interhemispheric synchrony (ASI) did not correlate to either HIE or NGT.

#### Correlations at 24 h of age

Altogether 13 (31%) EEG features correlated significantly to the HIE grade ([r]= 0.39−0.56). The most prominent findings included the background level (BSN, *r* = −0.43), intra- and interhemispheric measures of amplitude (aEEG, rEEG, PSD) as well as the same set of synchrony measures that showed clinical correlation already at 6 h of age (ASI, wPLI, cPSD, wPLI; see Fig. 3 for exact values). Six EEG features correlated to NGT ([r]= 0.36−0.49), including the EEG background (BSN, *r* = −0.36) and the same five synchrony measures that also correlated to the HIE grade (wPLI, cPSD). Only synchrony measures (*N* = 6) correlated to HINE ([r]= 0.39−0.61), while no features correlated to MRI or 2 years outcome scores.

#### Further observations

Additionally, we report the full correlation analysis (uncorrected for multiple corrections) both graphically in Fig. S3, and with numerical details in Supplementary Table S2. Comparisons across the full EEG feature matrix (Supplementary Table S2) shows that feature-outcome correlations are not randomly distributed. Instead, there are clearly identifiable groups of EEG features that persist over early hours (both 6 and 24 h of age), and between successive follow-up time points. Notably, several of these findings don’t pass correction for multiple comparison (see above) though they have been previously proposed as individual characteristics of clinical interest.^16,27,40,43–45^

## Discussion

Our results show that very early neonatal (a)EEG after perinatal asphyxia can provide objectively quantified computational EEG features with significant correlations to clinical outcome even in infants with only PA or mild-to-moderate HIE. These findings are in line with prior publications showing correlations between individual features and HIE grade or EEG backgrounds.^21,22,27,46^ In this work, the strongest clinical correlations were observed with features related to EEG amplitude or spatial synchrony, which can be intuitively related to the strength of cortical activity or the interareal communication in the cortico-cortical networks.

The present findings extend prior literature where comparable clinical correlations were only reported when the severe HIE is included;^25,29^ here we show that many EEG features can also exhibit robust clinical correlations within the milder HIE groups. The developmental sequelae after mild to moderate HIE have been well established, however the group of infants with mild HIE or perinatal asphyxia without HIE is still poorly understood. Early EEG-based measures could provide additional complementary tools for clinical decisions in the large group of patients with milder sequalae of perinatal asphyxia.^3,4,20,42,47,48^

Our EEG findings also suggest that the cortical activity shows graded relationship to the severity of HIE (Fig. 2b, c), which is fully compatible with the clinical experience that the natural distribution of conditions does not adhere to the discrete categories.^49^ The insufficient clinical discrimination by the current HIE grading is most obvious for the infants with milder sequelae ranging from the PA to HIE2.^4,20,47,48^ Early stratification or outcome prediction in these infants is inaccurate with the clinical assessment alone.

Our computational EEG feature set included an interpretative, deep learning -based background index BSN,^41,42^ because the early background recovery is a well-known characteristic used for distinguishing the clinically identified HIE grades and for predicting clinical outcomes.^32,33,42,50^ Results of the temporal dynamics of the BSN in the groups with different HIE grades (Fig. 2c) illustrate that accounting for postnatal age is essential when assessing the early cerebral recovery with (a)EEG for decision-making at different time points. Notably, our findings show that BSN value alone is significantly correlated to the HIE grade in infants ranging from PA to HIE2, although it does not correlate to their later clinical outcomes.

In the clinical practice it is a common experience that a trained human expert may visually perceive more than what is presented by discrete EEG background categories,^12,51^ which are typically used as benchmarks in clinical trials^52^ or aEEG classifications. Findings of the present study support the idea that the EEG signal can present information beyond what is visually discernible in the EEG signals, and that these EEG characteristics can be measured automatically to complement the traditional visual analyses. Our work shows further that computational EEG features could potentially be used for supporting objective definition of grades in the full spectrum of perinatal asphyxia.^49^

To conclude, our findings suggest that automated, EEG-derived computational measures hold promise for an early objective, quantitative, and automated assessment in the milder forms of perinatal asphyxia. Such measures underpin functional biomarkers for use in individualized therapeutic decisions and for benchmarking clinical trials and research. Moreover, computational EEG measures may provide effective translational bridges between clinical and preclinical experiments.

## Supplementary information

Supplementary Information

## Data Availability

The datasets generated during the current study are available from the corresponding author on reasonable request.
