# Supplementary material for: Quantitative EEG features during the first day correlate to clinical outcome in perinatal asphyxia
Source: Pediatr Res. 2024 May 14;97(1):261–7. doi: 10.1038/s41390-024-03235-y (PMC11798844; doi:10.1038/s41390-024-03235-y)
Supplement: Supplementary file 1 — Supplementary Information [file 41390_2024_3235_MOESM1_ESM.pdf]

Supplementary material

Quantitative EEG features during first day of life correlate to early clinical outcome in perinatal asphyxia

Anna Tuiskula*[a,b], Alexey S Pospelov[b,c], Päivi Nevalainen[b,d], Saeed Montazeri[b,c], Marjo Metsäranta[a,b], Leena Haataja[b,e], Nathan Stevenson[f], Anton Tokariev[b,c] and Sampsa Vanhatalo[b,c,d]

[a] Department of Pediatrics, Children's Hospital, University of Helsinki and Helsinki University Hospital, Helsinki, Finland

[b] BABA Center, Pediatric Research Center, University of Helsinki and Helsinki University Hospital, Helsinki, Finland

[c] Department of Physiology, University of Helsinki, Helsinki, Finland

[d] Department of Clinical Neurophysiology, Children's Hospital, HUS Diagnostic Center, and Epilepsia Helsinki, full member of ERN EpiCare University of Helsinki and Helsinki University Hospital, Helsinki, Finland

[e] Department of Pediatric Neurology, Children's Hospital, University of Helsinki and Helsinki University Hospital, Helsinki, Finland

[f] Brain Modelling Group, QIMR Berghofer Medical Research Institute, Brisbane, QLD, Australia.

A

	Total N	NGT	MRI	HINE	GMDS-III
PA	18	18	17	16	14
HIE1	10	10	10	10	6
HIE2	52	12	12	12	9
HIE3	11	0	0	0	0

B

	HIE grade	NGT	MRI	HINE	GMDS-III
HIE grade		0,69	0,49	-0,49	-0,11
NGT			0,40	-0,47	-0,31
MRI				-0,36	-0,27
HINE					0,13
GMDS-III					

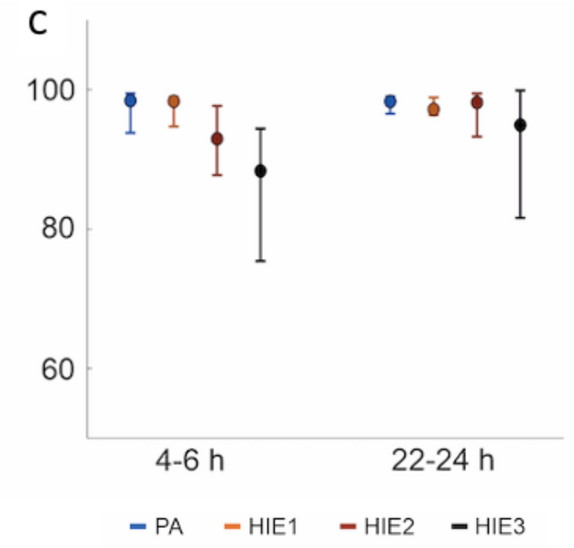

Figure S1. HIE groups, clinical correlations, and data availability.

(A) The numbers of patients from which the clinical outcomes data were collected.

(B) The correlations (Spearman's correlation coefficients) of HIE grades and clinical outcomes corrected for multiple comparisons. The colored values are statistically significant.

(C) The percentage of the EEG classified as clean in two time periods and four HIE grades, median and quartiles.

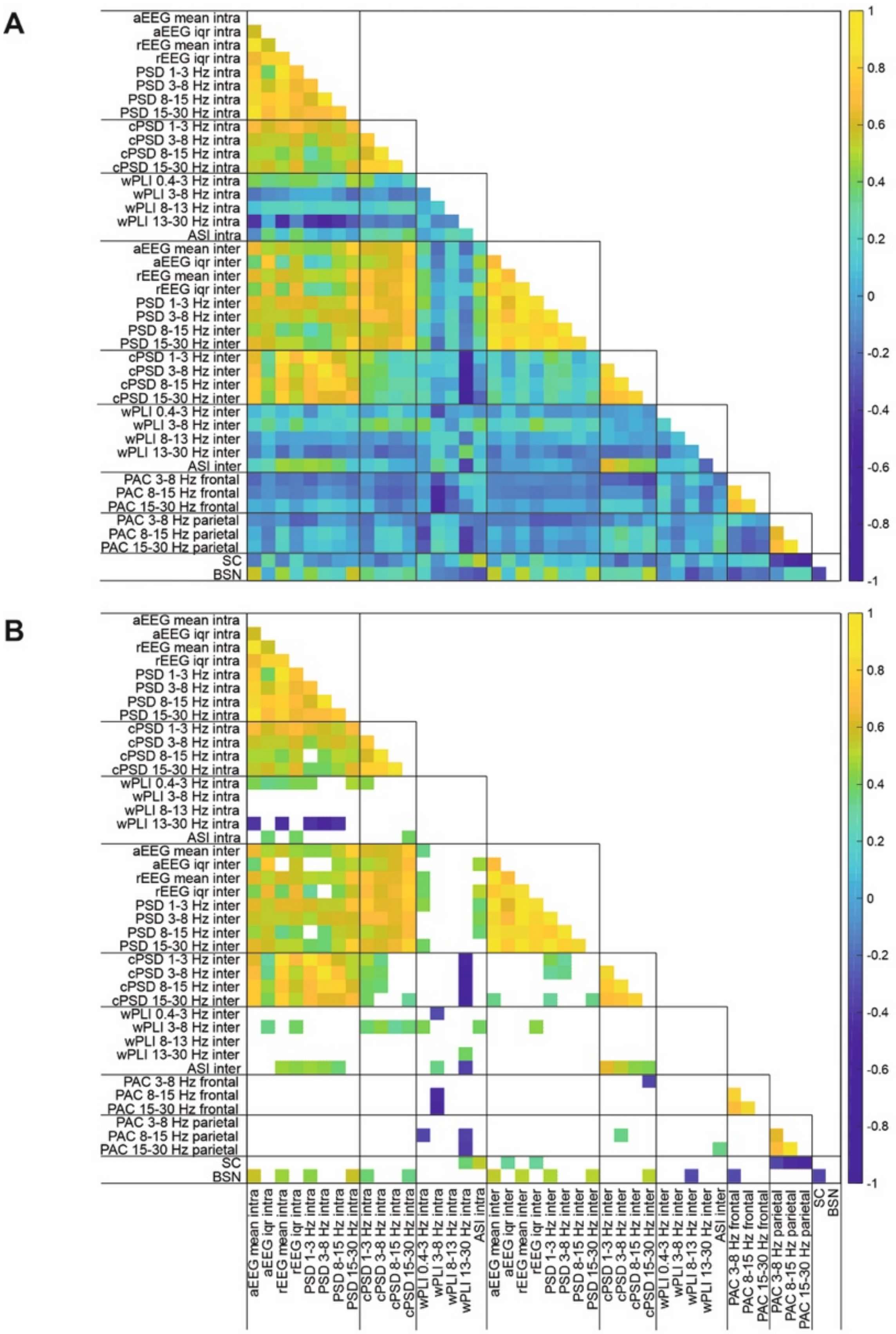
A
B
aEEG mean intra
aEEG iqr intra
rEEG mean intra
rEEG iqr intra
PSD 1-3 Hz intra
PSD 3-8 Hz intra
PSD 8-15 Hz intra
PSD 15-30 Hz intra
cPSD 1-3 Hz intra
cPSD 3-8 Hz intra
cPSD 8-15 Hz intra
cPSD 15-30 Hz intra
wPLI 0.4-3 Hz intra
wPLI 3-8 Hz intra
wPLI 8-13 Hz intra
wPLI 13-30 Hz intra
ASI intra
aEEG mean inter
aEEG iqr inter
rEEG mean inter
rEEG iqr inter
PSD 1-3 Hz inter
PSD 3-8 Hz inter
PSD 8-15 Hz inter
PSD 15-30 Hz inter
cPSD 1-3 Hz inter
cPSD 3-8 Hz inter
cPSD 8-15 Hz inter
cPSD 15-30 Hz inter
wPLI 0.4-3 Hz inter
wPLI 3-8 Hz inter
wPLI 8-13 Hz inter
wPLI 13-30 Hz inter
ASI inter
PAC 3-8 Hz frontal
PAC 8-15 Hz frontal
PAC 15-30 Hz frontal
PAC 3-8 Hz parietal
PAC 8-15 Hz parietal
PAC 15-30 Hz parietal
SC
BSN
1
0.8
0.6
0.4
0.2
0
-0.2
-0.4
-0.6
-0.8
-1

Figure S2. Correlations between EEG features.

(A) The heatmap of correlations for all pairs of the EEG features.

(B) The same heatmap with non-significant values omitted ($p>0.05$, no multiple comparison compensation). The data is computed for the median values at 22-24 hours of age in patients who have clinical outcomes available (N=40). The features are ordered by their characteristics to facilitate recognition of more correlating feature clusters. For instance, amplitude-related features have generally higher correlations, while the phase-related features are relatively uncorrelated. Notably, the background measure BSN exhibits significant correlations to individual, scattered features only.

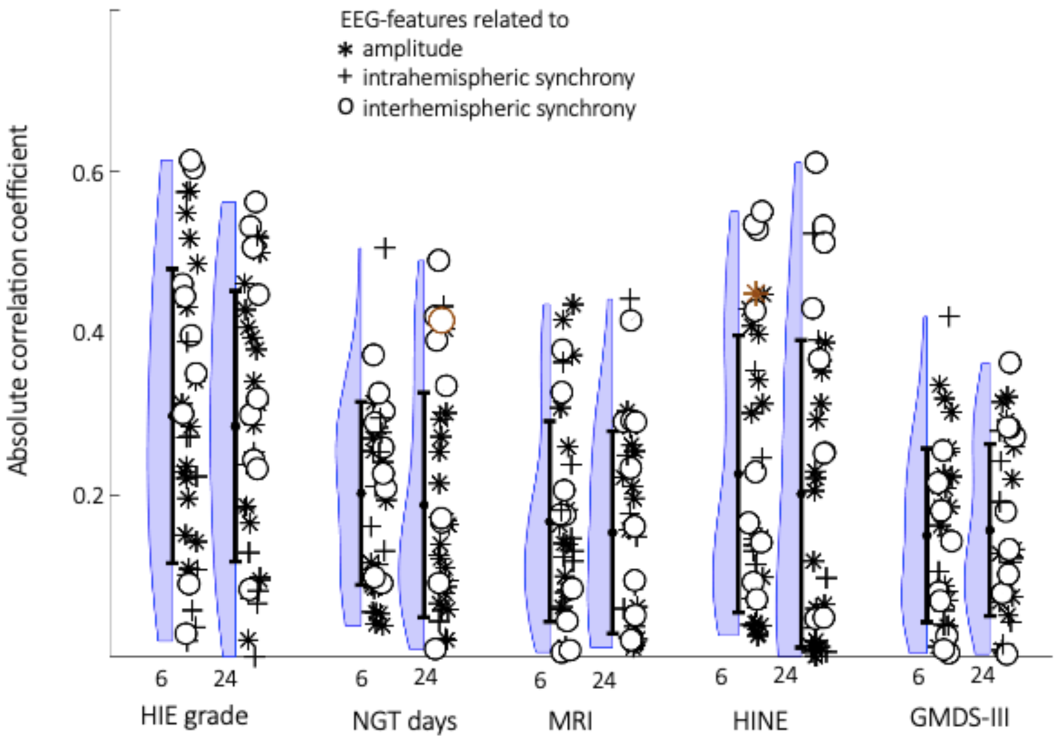

Figure S3. Correlations between individual EEG features and different clinical outcomes. Specific correlation coefficients (absolute value of Spearman r) are on the Y axis and different outcome modalities on the X axis. Each symbol represents one correlation between different EEG features and outcome modalities. Two different time points of EEG features (6h and 24h, respectively) have been distinguished. Note the wide variability in correlation strengths. Results from some features (PAC, SC, and BSN) are only shown in the Supplementary Table S2 as they do not fall into these thematic categories.

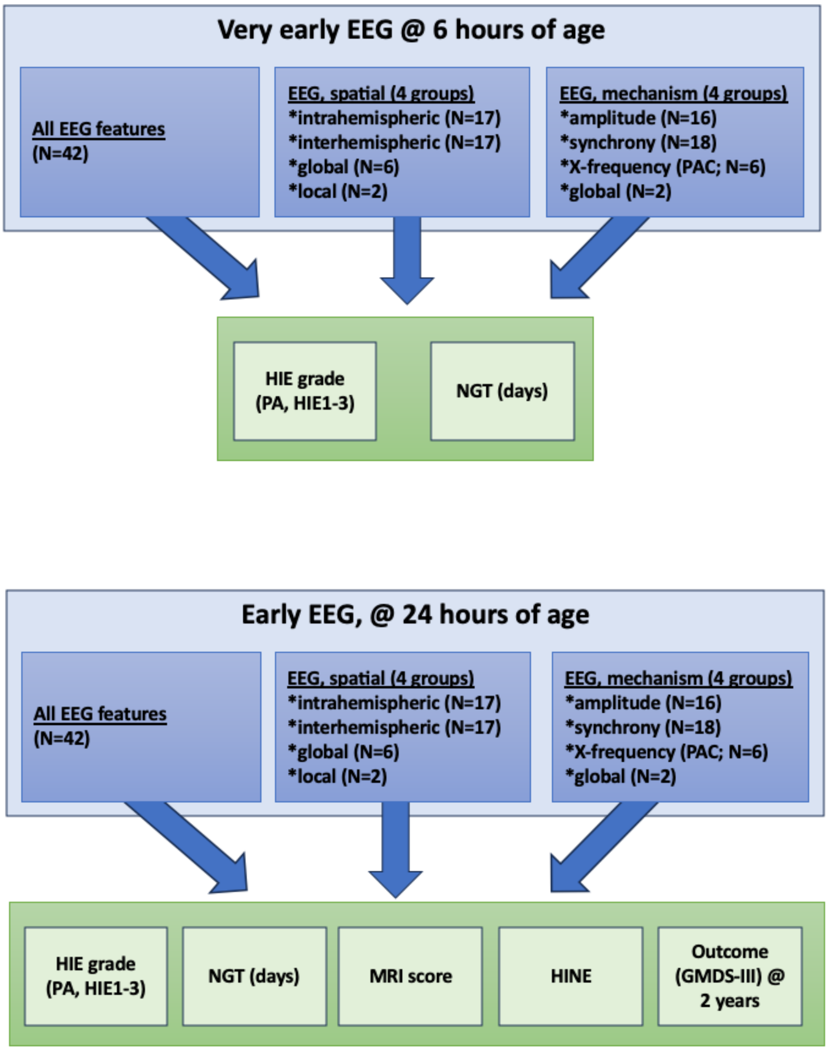

Figure S4. Graphical presentation of the statistical analysis strategy, indicating controlling for false discoveries using Benjamin-Hochberg method (i.e. correction for multiple comparisons). We used three different analytic approaches for each postnatal age. First, all the EEG features were correlated to the given clinical outcome (N=42 comparisons). Second, the EEG features were ordered spatially into four groups (N=2-17 comparisons per group). Third, the EEG features were ordered by neuronal mechanisms into four groups (N=2-18 comparisons per group).

		GLOBAL		INTRA																
		SC	BSN	aEEG iqr	rEEG iqr	aEEG mean	rEEG mean	PSD 1-3Hz	PSD 3-8Hz	PSD 8-15Hz	PSD 15-30Hz	cPSD 1-3Hz	cPSD 3-8Hz	cPSD 8-15Hz	cPSD 15-30Hz	wPLI 0.4-3Hz	wPLI 3-8Hz	wPLI 8-13Hz	wPLI 13-30Hz	ASI
6 hours	HIE	**0,55**	**-0,39**	**-0,55**		**-0,43**			**-0,58**	**-0,52**	**-0,49**								**0,39**	**0,58**
	NGT	**0,50**																		**0,51**
24 hours	HIE		**-0,43**	**-0,39**		**-0,50**		**-0,52**	**-0,46**		**-0,43**								**0,52**	
	NGT		-0,36																	
	MRI																			
	HINE																		**-0,52**	
	GMDS-III																			

		INTER																	LOCAL
		aEEG iqr	rEEG iqr	aEEG mean	rEEG mean	PSD 1-3Hz	PSD 3-8Hz	PSD 8-15Hz	PSD 15-30Hz	cPSD 1-3Hz	cPSD 3-8Hz	cPSD 8-15Hz	cPSD 15-30Hz	wPLI 0.4-3Hz	wPLI 3-8Hz	wPLI 8-13Hz	wPLI 13-30Hz	ASI	
6 hours	HIE									**-0,46**	**-0,60**	**-0,45**	**-0,61**	**0,40**					
	NGT																		
24 hours	HIE	-0,38				**-0,41**				**-0,51**	**-0,56**	**-0,45**	**-0,53**						
	NGT									-0,42		-0,42	-0,49						
	MRI																		
	HINE									0,43	**0,53**	**0,51**	**0,61**						
	GMDS-III																		

0.30-0.45
0.45-0.60
**0.60-**

Figure S5. Summary of significant correlation findings between spatially grouped EEG features and clinical outcomes for both 6h and 24h postnatal age. The findings are the same as shown in Fig 3A, but here the correction for multiple comparisons is done for spatially grouped EEG features. The matrix shows only findings that pass correction for multiple comparisons in each spatial group (global, intrahemispheric, interhemispheric, local). The bold fonts show corrections done for all N=42 EEG features together. Color indicates strength of correlation coefficient.

	Feature	Description	Technical details
Amplitude-related features	**aEEG mean**<br>**aEEG iqr**<br>**rEEG mean**<br>**rEEG iqr**	The set of features that characterize the peak-to-peak amplitude of the EEG.<br>iqr= interquartile range.	*Interhemispheric:* calculated from a P3-P4 derivation.<br>*Intrahemispheric:* average of values calculated from F3-P3 and F3-P4 derivations.<br>0.4-30 Hz
	**PSD**<br>=power spectral density	The features characterizes the power of the EEG in specific frequency band.	*Interhemispheric:* calculated from a P3-P4 derivation<br>*Intrahemispheric:* average of values calculated from F3-P3 and F3-P4 derivations<br>1-3 Hz, 3-8 Hz, 8-15 Hz 15-30 Hz
Synchrony - related features	**cPSD**<br>=cross power spectral density	The feature characterizes synchronicity of high-power time periods between two derivations in specific frequency band.	*Interhemispheric*: calculated for a pair of F3-P3 and F4-P4 derivations.<br>*Intrahemispheric*: average of values calculated for pairs of F3 vs P3 and F3 vs P4 channels.<br>1-3 Hz, 3-8 Hz, 8-15 Hz,15-30 Hz
	**wPLI**<br>=weighted phase lag index	The feature characterizes the phase synchrony between two derivations over time.	*Interhemispheric:* calculated for a pair of F3-P3 and F4-P4 derivations.<br>*Intrahemispheric*: average of values calculated for pairs of F3 vs P3 and F3 vs P4 channels.<br>0.4-3 Hz, 3-8 Hz, 8-13 Hz,13-30 Hz
	**ASI**<br>=activation synchrony index	The feature characterizes the amplitude-amplitude synchrony between two derivations.	*Interhemispheric:* calculated for a pair of F3-P3 and F4-P4 derivations<br>*Intrahemispheric:* average of values calculated for pairs of F3 vs P3 and F3 vs P4 channels<br>0.4-30 Hz
	**PAC**<br>=phase-amplitude coupling	The feature characterizes the synchrony of a phase of low- frequency component and the amplitude of high-frequency component of a specific frequency band.	*Frontal:* average of values calculated for F3 and F4 channels.<br>*Parietal:* average of values calculated for P3 and P4 channels.<br>3-8 Hz, 8-15 Hz, 15-30 Hz
Global features	**SC**<br>=suppression curve	The feature that characterizes the periods of suppressed EEG.	All channels combined 0.4-30 Hz.
	**BSN**<br>=brain state of the newborn	An interpretive, deep learning -derived measure of the EEG background activity	All channels combined 0.4-30 Hz.

Table S1. The EEG features used in the present study. The features were computed from one or two EEG signals, depending on their definition (see below). *Intrahemispheric* refers to measures from one side of the head only, while *interhemispheric* measures used EEG from both sides of the head.

	HIE grade		Time to full oral feeding		MRI		HINE		GMDS-III	
	4-6 hours	22-24 hours	4-6 hours	22-24 hours	4-6 hours	22-24 hours	4-6 hours	22-24 hours	4-6 hours	22-24 hours
aEEG mean intra	−0,55	−0,39	−0,31	−0,25	−0,44	−0,21	0,45	0,31	0,04	0,08
aEEG iqr intral	−0,10	−0,02	0,09	0,17	−0,14	0,04	0,15	−0,02	0,09	0,08
rEEG mean intra	−0,43	−0,50	−0,24	−0,29	−0,26	−0,30	0,43	0,39	0,08	0,05
rEEG iqr intra	−0,15	−0,17	0,12	−0,06	−0,16	−0,02	0,30	0,12	0,16	−0,01
PSD 1-3 Hz intra	−0,34	−0,52	−0,28	−0,41	−0,18	−0,25	0,41	0,37	0,07	0,01
PSD 3-8 Hz intra	−0,58	−0,46	−0,27	−0,27	−0,31	−0,26	0,45	0,35	0,21	0,29
PSD 8-15 Hz intra	−0,52	−0,31	−0,26	−0,12	−0,42	−0,28	0,34	0,29	0,19	0,32
PSD 15-30 Hz intra	−0,49	−0,43	−0,26	−0,22	−0,37	−0,22	0,40	0,23	0,01	−0,07
cPSD 1-3 Hz intra	0,04	−0,25	0,06	−0,09	0,06	0,06	−0,04	0,02	−0,07	−0,12
cPSD 3-8 Hz intra	0,02	−0,18	0,21	0,10	0,15	0,02	−0,13	0,00	0,11	−0,12
cPSD 8-15 Hz intra	−0,06	−0,10	0,16	0,09	0,12	0,01	−0,14	0,01	−0,06	−0,10
cPSD 15-30 Hz intra	−0,11	−0,24	0,11	−0,04	0,08	0,01	−0,11	0,01	−0,25	−0,28
wPLI 0.4-3 Hz intra	0,27	−0,13	0,13	−0,17	0,24	−0,09	−0,04	−0,04	−0,15	−0,10
wPLI 3-8 Hz intra	0,22	0,00	0,28	−0,06	0,13	0,15	−0,06	−0,10	−0,08	−0,24
wPLI 8-13 Hz intra	0,27	−0,07	0,25	−0,02	0,12	−0,18	−0,07	−0,01	−0,04	0,19
wPLI 13-30 Hz intra	0,39	0,52	0,30	0,43	0,18	0,44	−0,25	−0,52	−0,42	−0,04
ASI intra	0,58	0,08	0,51	0,13	0,36	0,25	−0,35	−0,39	0,04	−0,02
aEEG mean inter	−0,31	−0,38	−0,09	−0,14	−0,08	−0,09	−0,04	0,06	−0,26	−0,13
aEEG iqr inter	0,11	0,08	0,32	0,30	0,14	0,16	−0,31	−0,21	−0,22	−0,13
rEEG mean inter	−0,24	−0,34	−0,09	−0,07	−0,04	−0,01	−0,03	0,00	−0,32	−0,27
rEEG iqr inter	0,14	−0,10	0,19	0,11	0,08	0,20	−0,15	−0,22	−0,23	−0,32
PSD 1-3 Hz inter	−0,20	−0,41	−0,06	−0,16	−0,01	0,01	−0,04	0,05	−0,34	−0,26
PSD 3-8 Hz inter	−0,28	−0,31	−0,05	−0,09	−0,10	0,02	−0,03	0,01	−0,19	−0,10
PSD 8-15 Hz inter	−0,22	−0,19	−0,04	0,02	−0,06	0,02	−0,10	−0,06	−0,21	−0,08
PSD 15-30 Hz inter	−0,23	−0,29	0,04	−0,08	−0,05	−0,06	−0,14	−0,01	−0,30	−0,22
cPSD 1-3 Hz inter	−0,46	−0,51	−0,30	−0,42	−0,18	−0,23	0,53	0,43	0,08	0,08
cPSD 3-8 Hz inter	−0,60	−0,56	−0,29	−0,39	−0,33	−0,29	0,53	0,53	0,22	0,36
cPSD 8-15 Hz inter	−0,45	−0,45	−0,26	−0,42	−0,21	−0,29	0,43	0,51	0,03	0,27
cPSD 15-30 Hz inter	−0,61	−0,53	−0,33	−0,49	−0,38	−0,42	0,55	0,61	−0,01	0,18
wPLI 0.4-3 Hz inter	0,40	0,24	0,09	0,34	0,18	−0,02	−0,09	−0,05	−0,01	0,10
wPLI 3-8 Hz inter	0,35	0,08	0,37	0,17	0,01	0,29	−0,23	−0,37	0,14	−0,28
wPLI 8-13 Hz inter	0,09	−0,23	0,21	−0,01	−0,01	−0,16	0,07	−0,05	0,18	−0,28
wPLI 13-30 Hz inter	0,30	0,30	0,23	0,09	0,04	−0,05	−0,17	−0,25	0,07	0,00
ASI inter	0,03	−0,32	0,10	−0,17	0,08	−0,10	0,14	0,25	0,26	0,13
PAC 3-8 Hz frontal	−0,28	0,01	−0,07	0,05	0,00	−0,17	0,18	−0,22	0,40	0,14
PAC 8-15 Hz frontal	−0,26	−0,04	−0,16	−0,09	−0,24	−0,08	0,18	−0,05	0,59	0,26
PAC 15-30 Hz frontal	−0,30	−0,29	−0,22	−0,19	−0,18	−0,25	0,25	0,13	0,62	0,22
PAC 3-8 Hz parietal	−0,16	−0,02	−0,26	0,21	−0,12	−0,34	0,18	0,06	−0,07	0,02
PAC 8-15 Hz parietal	−0,27	−0,30	−0,27	−0,04	0,04	−0,37	0,26	0,37	−0,01	−0,10
PAC 15-30 Hz parietal	−0,25	−0,29	−0,28	−0,06	−0,15	−0,40	0,24	0,29	−0,08	−0,14
SC	0,55	0,18	0,50	0,06	0,31	0,20	−0,33	−0,36	−0,07	0,10
BSN	−0,39	−0,43	−0,26	−0,36	−0,13	−0,06	0,13	0,29	−0,08	−0,09

Table S2. The Spearman correlation coefficients between the HIE grade and clinical outcomes vs the EEG features. “Inter” and “intra” mean “interhemispheric” and “intrahemispheric” respectively. The values are colored based on the significance (no multiple comparison compensation): yellow: $p < 0.05$; peach: $p < 0.01$; orange: $p < 0.001$.

HIE grade	Therapeutic hypothermia	Electrographic seizures	Age at onset of seizures	Medication and doses	Time to full oral feeding
HIE2	no (onset of seizures >6h)	several	10h	phenobarbital x3 & levetiracetam x 3	10d
HIE2	no (onset of seizures >6h)	several	23h	phenobarbital x1	5d
HIE2	yes	several	23h	phenobarbital x1	7d
HIE2	yes	several	7h	phenobarbital x1	5d
HIE2	yes	several	14h	phenobarbital x1	11d

Table S3. Anticonvulsants given to infants in cohort 1. None of the infants with PA or HIE1 received antiseizure medications.
